# Pediatric patients with familially inherited sitosterolemia: Two case reports

**DOI:** 10.3389/fcvm.2022.927267

**Published:** 2022-08-16

**Authors:** Shun-Qing Su, Di-Sheng Xiong, Xiu-Mei Ding, Jin-An Kuang, Yue-Chun Lin

**Affiliations:** Department of Burn and Plast, Dalang Hospital of Dongguan, Dongguan, China

**Keywords:** sitosterolemia, ABCG5 gene, xanthoma, surgery heterozygous pathogenic, therapy

## Abstract

**Background:**

Sitosterolemia is a rare recessive genetic abnormality of hyperlipidemia; it is characterized by increased levels and accumulation of sitosterol in the plasma and local tissues.

**Case descriptions:**

The study subjects were two siblings (brother and sister) who had sitosterolemia with systemic multiple xanthomas as the main manifestation. The main clinical manifestations were hypercholesterolemia, premature atherosclerosis, arrhythmia, systemic multiple xanthomas, etc. After genetic testing, it was found that the patients had a compound heterozygous mutation of c.1324+1de1G in exon 7 and exon 9 of chromosome 2p21 of the adenosine triphosphate binding cassette transporter G family member *5(ABCG5)* gene; the mutation at c.904+1G>A was of maternal origin, and the mutation at c. 1324+1de1G was of paternal origin. The compound heterozygous mutation of these two genes led to a metabolic disorder of plant sterols *in vivo*.

**Conclusion:**

Sitosterolemia is an autosomal recessive disease that could be effectively controlled after dietary control and oral lipid-lowering therapy with Ezetimibe. Xanthomas, which affects function and appearance, could be surgically removed, and primary wound healing could be achieved.

## Introduction

Sitosterolemia is a rare, genetically abnormal hyperlipidemia; it is recessively inherited and characterized by increased levels and accumulation of sitosterol in the plasma and tissues. Clinical manifestations include xanthoma, early-onset atherosclerosis, angina pectoris, myocardial infarction, intermittent claudication, arthritis, arthralgia, and hemolysis (1). At present, there are >100 cases with sitosterolemia being reported with a detailed genetic diagnosis. Most Asian patients have adenosine triphosphate binding cassette transporter G family member *5(ABCG5)* gene mutations, while most Caucasian patients have *ABCG8* gene mutations (2). With the improvement in genetic diagnosis technology, relevant case reports and discussions have emerged in China (3, 4). Two cases of siblings (brother and sister) who had sitosterolemia with multiple xanthomas as the main manifestation were diagnosed and treated in Burn and Plast department; the details are reported as follows.

## Case report

### Case 1

The two included patients were school-age children, and the case 1 was the elder brother of the case 2. The parents are carriers of the mutated gene, but do not have the following discussed disease. The case 1 was a male patient aged 9 years old was admitted to Dalang Hospital of Dongguan (DHD) presented with masses in the buttocks and extremities that had been present for 4 years.

### Case history

As detailed in Table 1, In July 2017, the patient visited a hospital in Guangzhou (Guangzhou Women and Children's Medical Center, GWCMC) due to “masses in the limbs and buttocks” and was examined; multiple xanthomas were found in the buttocks and joints of the limbs. The laboratory results (Table 2) were as follows: serum total cholesterol (TC) = 15.13 mmol/L, serum triglyceride (TG) = 1.95 mmol/L, high-density lipoprotein cholesterol (HDL-C) = 1.99 mmol/L, low-density lipoprotein cholesterol (LDL-C) = 14.04 mmol/L, apolipoprotein B (Apo B) = 3.08 g/L, apolipoprotein E (APOE) = 95.4 mg/L, and free fatty acids (FFAS) = 1.4 mmol/L. After multiple re-examinations, the above blood lipid parameters were all elevated. The patient was diagnosed with “familial hypersteroidemia, xanthoma”. The results of the mass biopsy indicated xanthoma. Lipid-lowering treatment was administered, but the masses in the limbs and buttocks gradually increased.

**Table 1:** Basic information of Case 1 from onset to date.

**Date**	**Clinical sign**	**Medical institution**	**Diagnosis**	**Treatment**
July 2017	Masses in the limbs and buttocks	GWCMC	Hypercholesterolemia, multiple lower extremity tumors	Levocarnitine orally
April 2020	Multiple xanthomas on the skin and arrhythmia for more than 2 years	FAHSU	Sitosterolemia, multiple arterial stenosis	Ezetimibe orally, diet control (reduce phytosterol intake)
July 2021	Xanthoma at the bilateral buttocks	DHD	Sitosterolemia, multiple arterial stenosis, multiple xanthomas	Ezetimibe orally, surgery

*GWCMC, Guangzhou Women and Children's Medical Center; FAHSU, Department of Pediatrics of the First Affiliated Hospital of Sun Yat-sen University; DHD, Dalang Hospital of Dongguan*.

**Table 2:** Blood lipid levels before and after lipid-lowering treatment.

	**2017-7 (GWCMC)**	**2020-4 (FAHSU)**	**2021-7 (DHD)**
TC (mmol/L)	15.13 (3.4–5.2)	4.5 (2.8–5.18)	6.19 (3.4–6.5)
TG (mmol/L)	1.95 (0.23–1.7)	0.57 (023–1.71)	1.39 (0.34–1.70)
HDL-C (mmol/L)	1.99 (0.88–1.81)	0.84 (0.96–1.15)	1.15 (1.0–2.20)
LDL-C (mmol/L)	14.04 (0–3.37)	3.05 (0–3.10)	2.85 (0–3.10)

*Data of the measured parameters were presented as the result (normal range). TC, total cholesterol; TG, triglyceride; HDL-C, high-density lipoprotein cholesterol; LDL-C, low-density lipoprotein cholesterol*.

In April 2020, the patient was admitted to the Department of Pediatrics of the First Affiliated Hospital of Sun Yat-sen University (FAHSU) due to “multiple xanthomas on the skin and arrhythmia for more than 2 years”. The results of the physical examinations were as follows: Temperature (T) = 37°C, Pulse (P) = 110 beat per minute (bpm), Respiration (R) = 24 times/min, blood pressure (BP) of the right upper limb = 84/65 mmHg, BP of the right lower limb = 102/64 mmHg, BP of the left upper limb = 95/60 mmHg, BP of the left lower limb = 96/48 mmHg, and body weight = 19.6 kg. Physical examination: the physical development was retarded, with multiple nodules all over the body; the nodules were yellowish-brown in color, soft in texture, round or oval in shape with clear borders, and obvious at large joints. There was no tenderness in the nodules or swelling and pain in the joints. Xanthelasmas of the bilateral media upper eyelid was present. The heart rate (HR) was 110 bpm, the cardiac rhythm was irregular, and premature beats could be heard. The heart sounds were strong, and no murmurs could be heard in any valve area. The laboratory results were as follows: TC = 4.5 mmol/L, TG = 0.57 mmol/L, HDL-C = 0.84 mmol/L, LDL-C = 3.05 mmol/L, serum C-reactive protein = 41.78 mg/L, erythrocyte sedimentation rate = 81 mm/h, interleukin 6 = 23.26 pg/mL, albumin = 38.2 g/L. Arterial color Doppler ultrasonography: signs of Takayasu's arteritis, stenosis of the initial segment of the subclavian artery with partial type stealing, bilateral common carotid artery stenosis, thickening, uneven dilatation, stenosis of the abdominal aortic wall, and proximal narrowing of the bilateral renal arteries. The electrocardiogram (ECG) showed frequent junctional premature beats, paroxysmal supraventricular tachycardia, and occasional premature ventricular contractions.

In April 2020, gene detection of hyperlipidemia was conducted in the Guangzhou KingMed Diagnostics Center. The results were as follows: a splice mutation at the intron 7 and intron 9 of chromosome 2p21 in the *ABCG5* gene. The mother and father of the patient carried the heterozygous variant at intron 7 and the heterozygous variant at intron 9, respectively, in the *ABCG5* gene (Table 3).

**Table 3:** Whole-exome sequencing results.

	**Gene**	**Chromosome location**	**Reference transcript**	**Location**	**At the cDNA level**	**At the protein level**	**Status**	**The classification of the variant**	**Father**	**Mother**
Case 1	*ABCG5*	2p21	NM_022436.2	Intron7	c.904+1G>A	p.?	Heterozygous	Pathogenic	Undetected	Heterozygous carrier
Case 1	*ABCG5*	2p21	NM_022436.2	Intron9	c.1324+1de1G	p.?	Heterozygous	Pathogenic	Heterozygous carrier	Undetected
Case 2	*ABCG5*	2p21	NM_022436.2	Intron7	c.904+1G>A	p.?	Heterozygous	Pathogenic	Undetected	Heterozygous carrier
Case 2	*ABCG5*	2p21	NM_022436.2	Intron9	c.1324+1de1G	p.?	Heterozygous	Pathogenic	Heterozygous carrier	Undetected

*It was detected that the subject carried two heterozygous pathogenic variants in the ABCG5 gene, the variants were located on different alleles, and the parents were carriers without the disease. The experiment No. of Case 1was NP22S0624, and Case 2 was VP22D01451*.

The diagnosis was as follows: (1) hereditary sitosterolemia; (2) multiple arterial stenosis; (3) arrhythmia; and (4) growth retardation (moderate). Ezetimibe was administered for lipid lowering, and clopidogrel bisulfate tablets (Talcom) were adopted for anticoagulation therapy. The masses on the bilateral buttocks became soft, but the shrinkage was not obvious.

### Examination results

In July 2021, the patient was admitted from the outpatient clinic (Dalang Hospital of Dongguan, DHD) with the complaint of “xanthoma at the bilateral buttocks”.

#### Physical examination

**Figure 1:**
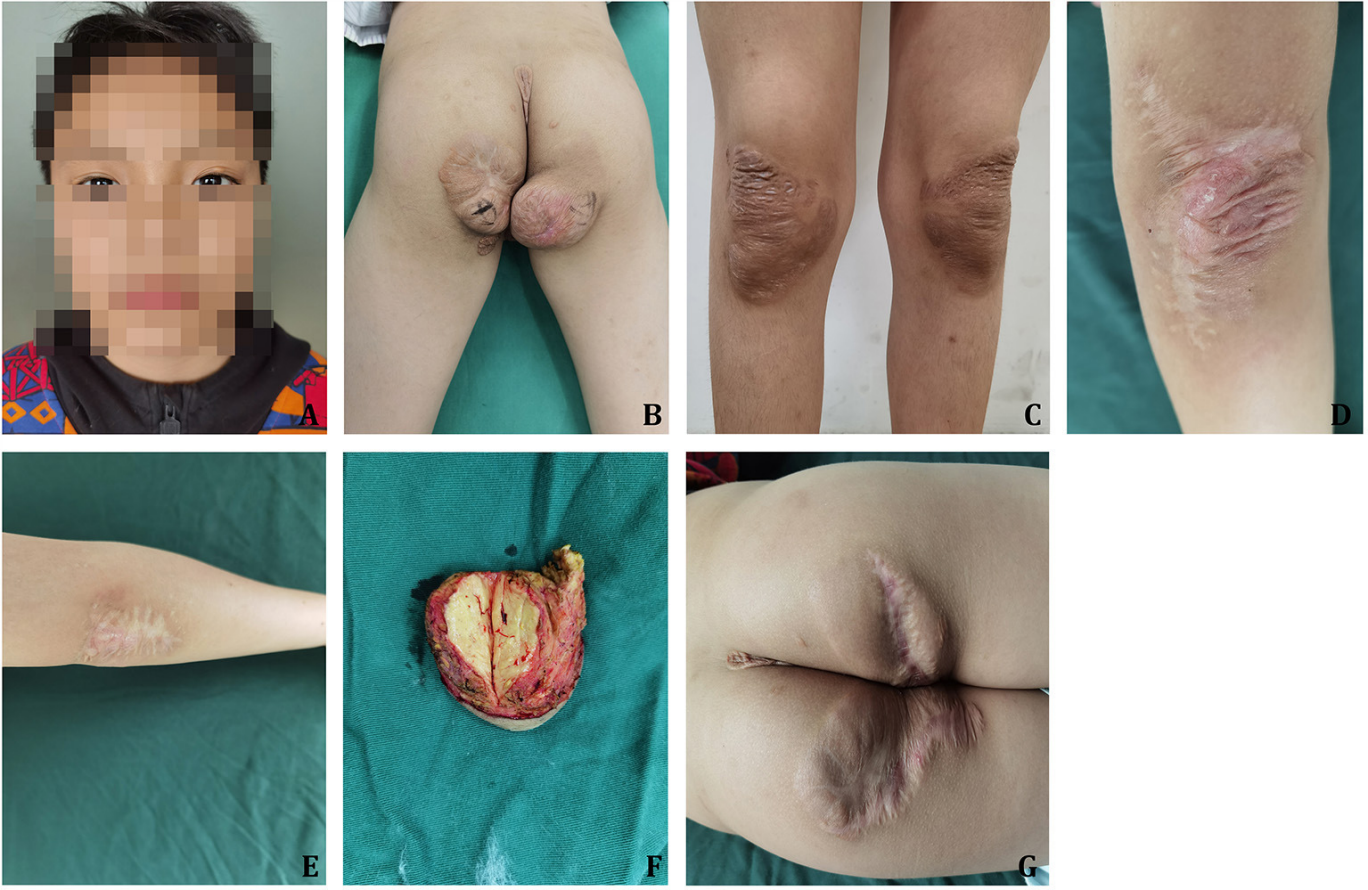
Case 1. **(A)** Xanthelasmas of the bilateral eyelids. **(B)** Xanthoma at the buttocks. **(C)** Partial shrinkage of tumor in the bilateral knee joints after lipid-lowering therapy. **(D)** One year after resection of xanthoma at the left elbow. **(E)** One year after resection of xanthoma at the right elbow. **(F)** The section of xanthoma at the time of resection. **(G)** Half a year after resection of xanthoma at the bilateral buttocks.

The results of physical examinations were as follows: T = 36.2°C, P = 84 bpm, R = 20 times/min, and BP = 102/53 mmHg. Xanthelasmas of the bilateral upper eyelids was present (Figure 1A). There were 7 × 8 and 8 × 8 cm round masses present in the bilateral buttocks, respectively (Figure 1B); these which protruded to the body surface without ulceration. Partial shrinkage of the mass at the knee joint after drug treatment was present (Figure 1C). A surgical excision biopsy had been conducted previously at the bilateral elbow joints, with postoperative scars (Figures 1D,E).

#### Other examination

**Figure 2:**
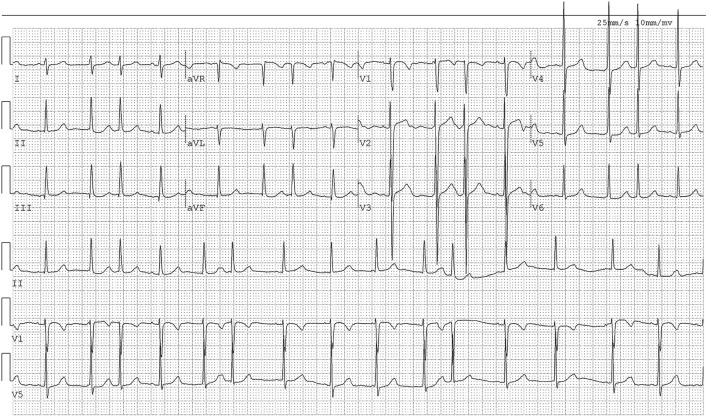
The ECG of Case 1. The ECG showed sinus arrhythmia and frequent premature atrial contractions.

ECG showed sinus arrhythmia and frequent premature atrial contractions (Figure 2). The laboratory results (Table 2) were as follows: TC = 6.19 mmol/L, TG = 1.39 mmol/L, HDL-C = 1.15 mmol/L, LDL-C = 2.85 mmol/L.

### Therapeutic intervention and outcomes

After routine examination, surgical resection was conducted under general anesthesia. The mass was brownish-yellow nodule-like, mostly distributed in the joints of the limbs and parts that were susceptible to pressure and friction, with clear boundaries, soft texture, and no tenderness; the mass was incised at 0.5 cm along the boundary of the mass and peeled off with an electric knife, and it was found that the mass was located at the superficial fascia, without obvious capsule, is pale yellow on the cut surface and tough in texture (see Figure 1F). It is seen under the microscope under the dermis, which is composed of sheets of foam-like histiocytes, a small number of multinucleated macrophages and proliferating fibrous tissue. The postoperative pathological results were tuberous xanthoma, and the patient achieved primary wound healing after the operation.

Lipid-lowering and anticoagulation therapy was continued after discharge, and no recurrence was observed during the 6-month follow-up (Figure 1G). The laboratory results in March 2022 were as follows: TG = 0.84 mmol/L, TC = 6.61 mmol/L, HDL-C = 1.52 mmol/L, LDL-C = 3.81 mmol/L, Apo B = 1.41 g/L. And the ECG results of this time were unchanged from the previous.

### Case 2

A female patient was aged 7 years. The patient was admitted in January 2022 due to “bumps in both elbows for more than 2 years”. The family complained of the findings of a mass in the bilateral elbow joint >2 years ago; this gradually grew without pain, ulceration, or restriction of elbow movement. The patient was hospitalized from the outpatient clinic with the chief complaint of “masses in the bilateral elbow joint” for surgical treatment.

### Case history

Molecular hereditary tests were conducted in March 2020 (Guangzhou KingMed Diagnostics Center). The result was a splice mutation at intron 7 and intron 9 on chromosome 2p21 of the *ABCG5* gene (Table 3); this was consistent with the results of Case 1.

### Examination results

#### Physical examination

**Figure 3:**
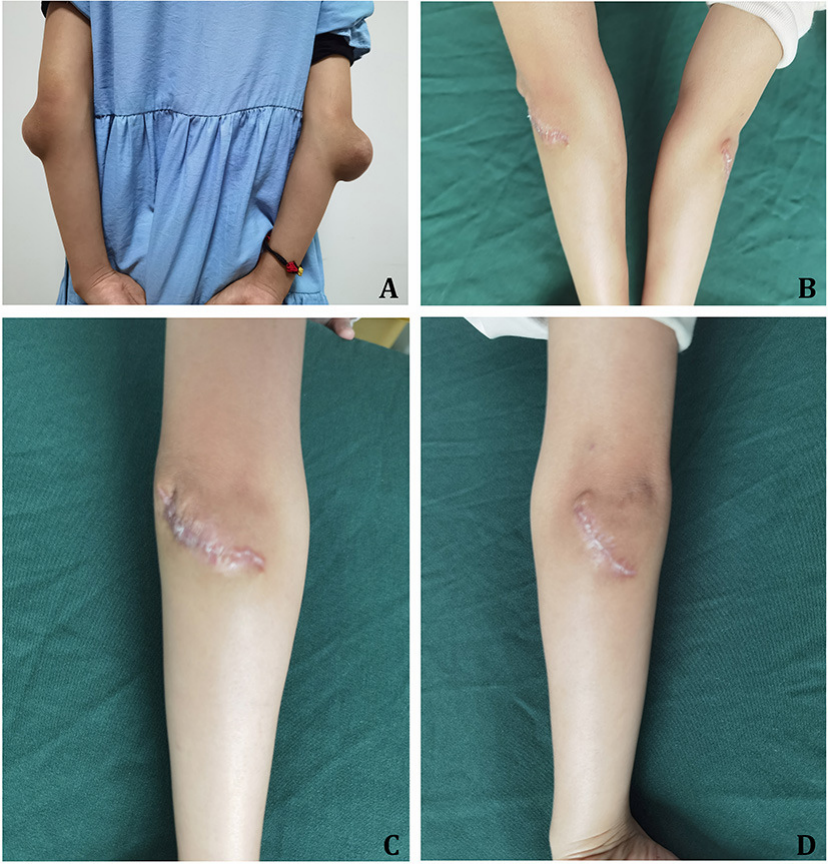
Case 2. **(A)** Xanthoma in the bilateral elbow before the resection. **(B–D)** Three months after the resection of xanthoma at the bilateral elbows.

The results of the physical examination were as follows: T = 36.3°C, P = 87 bpm, R = 20 times/min, and BP = 105/58 mmHg. The patient was clear-minded, with a normal development and moderate nutritional status. The patient walked to the hospital. The lungs were clear without any rales. The apical beat position was normal, and there was no lifting beat. The HR was 87 bpm, and the rhythm was irregular. No murmur could be heard in the auscultation area of any valve. On the extensor sides of the bilateral elbow joints, bulges of approximately 7.5 × 7.5 cm (right) and 3.7 × 2.2 cm (left) were observed. The surface skin was unruptured, the texture was tough, and the surrounding boundary was still clear with no tenderness. There was no swelling or pain in the joints as well as no movement restriction (Figure 3A). There was no xanthelasma on the eyelids.

#### Other examination

**Figure 4:**
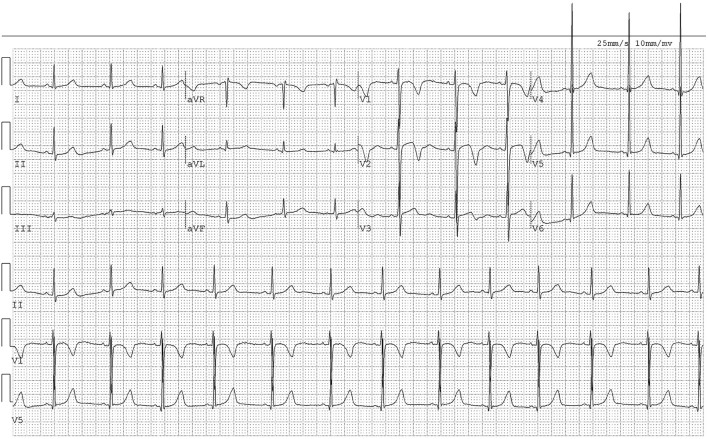
The ECG of Case 2. The ECG showed sinus arrhythmia.

The laboratory results were as follows: TG = 0.72 mmol/L, TC = 7.01 mmol/L, HDL-C = 1.65 mmol/L, LDL-C = 3.94 mmol/L, HDL-C/LDL-C ratio = 0.42, serum Apo A1 = 1.42 g/L, and serum Apo B = 1.57 g/L. The ECG showed sinus arrhythmia (Figure 4). The results of the color Doppler ultrasonography revealed that the inner diameters of the bilateral common carotid arteries, internal carotid arteries, and external carotid arteries were normal, and that the left and right carotid arteries were irregularly thickened, with a thicknesses of 1.5 mm and 1.2 mm at the thickest parts, respectively.

### Therapeutic intervention and outcomes

The diagnosis was sitosterolemia, and the patient was treated with Ezetimibe for long-term lipid lowering. After admission, bilateral elbow tumor resection was performed, and the postoperative pathological results showed xanthoma.

The patient was followed up with for 3 months post-operation; the incision healed well (Figures 3B–D). The laboratory results in March 2022 were as follows: TG = 0.54 mmol/L, TC = 7.26 mmol/L, HDL-C = 1.85 mmol/L, LDL-C = 4.22 mmol/L, Apo B = 1.59 g/L. And the ECG results of this time were unchanged from the previous.

The parents of the two pediatric patients had normal cholesterol levels and no signs of xanthomas or atherosclerotic plaques.

## Results of hereditary tests

### Test result interpretations

Table 3, Whole-exome sequencing results: There existed a compound heterozygous mutation of c.1324+1de1G in Intron 7 and Intron 9 of chromosome 2p21 of the *ABCG5* gene; the mutation at c.904+1G>A was of maternal origin, and the mutation at c.1324+1de1G was of paternal origin. No reports concerning these mutations have been conducted so far, and software analysis suggested a pathogenic mutation. The variant, *ABCG5* 2p21 NM_022436.2 Intron 7 p.? c.904+1G>A, was a splice mutation and was expected to cause changes in the splice site. It has been reported in relevant literature that this variant has been detected in patients with sitosterolemia. It was not included in the ESP6500siv2-ALL and Thousand Genomes (1000g2015aug-ALL) databases; however, it was included in the dbSNP147 database (5). The variant, *ABCG5* 2p21 NM_022436.2 Intron 9 p.? c.1324+1delG, was a splice mutation and was expected to cause changes in the splice site. It was not included in either the HGMD database or the ESP6500siv2-ALL, Thousand Genomes (1000g2015aug-ALL), and dbSNP147 database. Preliminary analysis *via* a bioinformatics software showed that the mutation significantly affected mRNA splicing, and a heterozygous pathogenic variant was detected on the trans allele of this variant. Taken together, this variant was considered a pathogenic variant. Preliminary analysis by bioinformatics software showed that it significantly affected mRNA splicing. Taken together, this variant was considered a pathogenic variant.

## Discussion

Sitosterolemia, also known as phytosterolemia, is a very rare sterol metabolism-related and autosomal recessive disorder caused by mutations in genes including *LDLR, PCSK9, APOE, ABCG* family gene, etc. The encoded products of *ABCG5/ABCG8* are involved in plant-derived sterol metabolism. In 2000, Berge et al. (4) found that defects in chromosome 2p21 lead to sitosterolemia. This pair of genes is highly expressed in liver and proximal small intestinal epithelial cells. There appears to be no correlation between the clinical phenotype and genotype, and there exist no significant differences in the clinical manifestations among patients with different genotypes (6). The incidence of the disease is estimated to be approximately 1 in 200,000 based on gene frequencies (7).

The *ABCG5/ABCG8* gene encodes a sterolin protein, which is involved in the metabolism of plant-derived sterols. Mutations in either gene may lead to increased intestinal absorption of plant sterols and decreased biliary excretion, resulting in increased levels of blood plant sterol (including sitosterol, campesterol, and stigmasterol) (8). In the normal human body, approximately 50% of dietary cholesterol is absorbed, while the absorption of plant sterols is <5%. The *ABCG5/ABCG8* heterodimeric transport protein can preferentially export free sterols from hepatocytes or intestinal epithelial cells to the lumen to promote excretion; thus, the plasma concentration of plant sterols is very low (4). Currently, different types of mutations in the *ABCG5* gene have been described in relevant literature; for example, the mutations in the c.1336C>T locus are the most common (9, 10). However, mutations in the c.904+1G>A and c.1324+1de1G loci of the *ABCG5* gene in the present cases had not yet been reported on. Generally, carriers of pathogenic variants do not develop into patients. If both parents are carriers, there is a 25% chance that their child will be affected. Other relatives of the patient's parents are also at risk of carrying the same pathogenic variant.

Sitosterolemia is a rare recessive genetic disorder of lipid metabolism. The age of onset is generally younger. The clinical symptoms are multiple xanthomas, atherosclerosis, angina pectoris, myocardial infarction, or accompanied by hemolysis. anemia, arthritis, etc. It displays obvious family relationship, homozygous disease, blood biochemical tests can be normal or increased plasma cholesterol, plasma LDL increased, Apo-B increased, HDL decreased, Apo-A1 decreased. The most diagnostic significance is significantly increased plasma phytosterols (mainly Sitosterol). When it is found that patients with the above clinical symptoms and their plasma cholesterol levels are normal or slightly increased, it should be considered suspected of this disease, and their plasma phytosterol levels and family investigations can be determined to confirm the diagnosis.

Sitosterolemia with a predominant presentation of xanthoma is easily misdiagnosed as familial hypercholesterolemia (FH). The clinical manifestations of sitosterolemia and FH are similar. But in biochemical characteristics, pathogenesis and genetic characteristics are different, FH is autosomal dominant inheritance, sitosterolemia is recessive inheritance. If early intervention and treatment are not timely, the prognosis is poor. Hence, differential diagnosis is necessary. The two diseases differ in pathogenesis, biochemical characteristics, and genetic features. Familial hypercholesterolemia is an autosomal dominant hereditary disease, and the related gene is the low-density lipoprotein receptor (LDLR), which encodes the LDLR and is involved in cholesterol metabolism. Among them, heterozygous mutations cause an early onset of atherosclerosis, xanthoma, myocardial infarction, etc. in adults, while homozygous mutations cause severe atherosclerosis in early childhood (11). In addition to cardiovascular disease, patients with FH may experience recurrent episodes of arthritis and tenosynovitis; however, these are without hematologic abnormalities (12).

Atherosclerosis of sitosterolemia is characterized by atherosclerosis of the coronary arteries or aorta that occurs in young adults, and atherosclerosis is accelerated, especially affecting young men. Several teenage boys have been reported to have died of acute myocardial infarction caused by extensive coronary atherosclerosis, the youngest of whom was an l3-year-old boy who had four homozygous siblings (13). An l7-year-old boy was reported with symptoms of progressive angina pectoris, and the ECG exercise test showed coronary hypoperfusion, and he died suddenly of acute myocardial infarction during exercise. The autopsy found that 60% of the left anterior descending coronary artery was occluded, and a small infarct was found in the myocardium (14). The 2 patients we reported had systemic multiple xanthoma as the main manifestation. From the onset to the diagnosis of the disease, they had been treated with lipid-lowering therapy, but the intake of phytosterols was not paid attention to the diet control, so the disease progressed. In case 1, segmental stenosis of left and right coronary arteries, bilateral common carotid artery stenosis, abdominal aortic wall thickening, heterogeneous dilation, stenosis, bilateral proximal renal artery stenosis, stenosis at the opening of the celiac trunk and superior mesentery artery were found in April 2020. Case 2 was young and had no symptoms of angina pectoris, only had bilateral carotid intima thickening. After the diagnosis, the targeted oral lipid-lowering drug ezetimibe was given, and vegetable oil, wheat germ, nuts, chocolate, shellfish, algae, etc. By the time of consultation in July 2021, multiple xanthomas had partially shrunk, and multiple arterial stenosis had not progressed significantly.

The main concerns of the patient's parents were whether the blood lipid is controlled to the normal levels or close to normal, whether there is recurrence after the operation, whether the incision forms a hypertrophic scar, etc. The purpose of the operation is to remove the mass and restore normal body signs. The tumor in Case 1 was located at the buttock's ischial tuberosity, and the tumor in Case 2 was at the extensor side of bilateral elbow joints, both of which were prone to pressure and friction. There was no hypertrophy or hypertrophy of postoperative incision scars, and no pressure ulcers occurred. Therefore, the surgical results were satisfactory.

Sitosterolemia is an autosomal recessive disorder. In addition to sitosterol accumulation, cholesterol is also elevated to some extent in patients with sitosterolemia. The 2 cases in the present study had hypercholesterolemic manifestations; the main clinical manifestations were atherosclerosis and xanthoma. The slow conversion and excretion of sitosterol to the liver and reduced fecal elimination might cause the expansion of the sitosterol “pool” in the body, followed by an increase in LDL intake (*via* LDLR); this may eventually lead to the deposition of sitosterol in the tissues and cause xanthoma. The clinical features of sitosterolemia in children mainly include skin/tendon xanthoma, arthralgia, red blood cell hemolytic anemia, thrombocytopenia, splenomegaly, and hyperphytosterolemia. However, some children may only show abnormal blood system without typical xanthoma. Jamwal et al. reported 3 children from an Indian family who presented with stomatocytosis, anemia, giant thrombocytopenia, growth arrest, and no xanthoma, and were later diagnosed with sitosterolemia caused by a homozygous mutation in the ABCG5 gene by genetic analysis (15).

The main pathological mechanism is the excessive absorption and slow excretion of plant sterols and the insufficient synthesis of endogenous cholesterol due to the inadequate activity of hydroxymethylglutaryl coenzyme A (HMG-CoA) reductase; hence, the key to reducing the concentration of sitosterol in blood and tissue is to reduce absorption and increase excretion.

Diet control, administration of lipid-lowering drugs, and intestinal bypass surgery are the main methods currently adopted in the treatment of sitosterolemia (16–18). Ezetimibe, a new type of cholesterol-lowering drug, is a selective cholesterol absorption inhibitor, which mainly blocks the exogenous absorption pathway of cholesterol by inhibiting intestinal cholesterol absorption by acting on cholesterol transporters. After strict dietary control and the administration of bile acid sequestrants, the xanthoma can be partially reduced, with significant improvement in the lipid indicators. Xanthoma affecting the appearance and function might be surgically removed. Continued administration of Ezetimibe tablets after surgery might inhibit tumor recurrence and the accumulation of intimal lipid plaques.

## Data availability statement

All data generated or analyzed during this study are included in this article. Further enquiries can be directed to the corresponding author.

## Ethics statement

Written informed consent was obtained from the minor's legal guardian/next of kin for the publication of any potentially identifiable images or data included in this article.

## Author contributions

S-QS and D-SX: conception and design of the research. D-SX: acquisition of data and critical revision of the manuscript for intellectual content. S-QS and X-MD: analysis and interpretation of the data. J-AK and Y-CL: statistical analysis. S-QS: writing of the manuscript. All authors read and approved the final draft.

## Conflict of interest

The authors declare that the research was conducted in the absence of any commercial or financial relationships that could be construed as a potential conflict of interest.

## Publisher's note

All claims expressed in this article are solely those of the authors and do not necessarily represent those of their affiliated organizations, or those of the publisher, the editors and the reviewers. Any product that may be evaluated in this article, or claim that may be made by its manufacturer, is not guaranteed or endorsed by the publisher.
